# Immersive 3D vs Physical Simulation in Training Novices for Laser-Based Endourological Procedures: Protocol for a Randomized Controlled Trial

**DOI:** 10.2196/94628

**Published:** 2026-10-06

**Authors:** Joséphine-Charlotte Coleman, Matthias J Witti, Natalia Wirtz, Maximilian Aumiller, Adrian Rühm, Ronald Sroka, Tamira Büttner, Benedikt Duckworth-Mothes, Matthias Stadler

**Affiliations:** 1 Institute of Medical Education, LMU University Hospital, LMU Medizin, Ludwig-Maximilians-Universität Munich, Pettenkoferstraße 8a, Munich, 80336, Germany, 49 440057209; 2 Department of Medicine IV, LMU University Hospital, LMU Medizin, Ludwig-Maximilians-Universität München, Munich, Germany; 3 Laser-Forschungslabor, LIFE (Laser- und Immunologie-Forschungs-Einrichtungen) Center, LMU University Hospital, LMU Munich, Munich, Bavaria, Germany; 4 Department of Urology, LMU University Hospital, LMU Munich, Munich, Bavaria, Germany; 5 medverse GmbH, Munich, Bavaria, Germany; 6 Institut für klinische Anatomie und Zellanalytik, Universitätsklinikum Tübingen, Tübingen, Baden-Württemberg, Germany

**Keywords:** simulation, virtual reality, physical simulator, endourology, randomized controlled trial

## Abstract

**Background:**

Simulation-based learning constitutes a valuable component of medical training, with immersive 3D simulations and physical simulations being commonly used. Immersive 3D simulation training has been proven to enhance procedural skills and their transfer, whereas physical simulators allow for haptic feedback. However, direct comparative evidence between these 2 simulation modalities is scarce and often does not address cognitive, motivational, and perceived learning outcomes, which also have an effect on actual learning outcomes.

**Objective:**

The objective of this study is to evaluate whether immersive 3D simulation (a primarily screen-based, PC- or tablet-delivered simulator with optional virtual reality elements) can serve as a complementary or preparatory modality to physical simulator training when teaching novice trainees endourological laser-based procedures. Specifically, we investigate whether pretraining on an immersive 3D simulator facilitates the transfer of procedural knowledge and technical skills to the physical simulator, alongside its effects on cognitive load, motivation, and perceived learning.

**Methods:**

We will conduct a single-center, 2-group randomized controlled trial with 70 medical students who are currently in the clinical phase of their undergraduate medical studies. Each participant will complete one training session on either the immersive 3D simulation or the physical simulator, followed by one posttraining test on the physical simulator. Technical performance will be assessed by 2 experts using an objective structured assessment of technical skills–based composites, a critical error checklist as an indicator of safety, and time to task completion as an indicator of efficiency. Furthermore, we will measure motivation, cognitive load, and perceived learning. To analyze group differences in the primary and secondary outcomes, we will use independent-sample *t* tests (1-tailed), with nonparametric or Welch-corrected alternatives in cases in which assumptions are violated, and regression analyses to examine associations between the secondary outcomes and technical performance.

**Results:**

Recruitment will start in September 2026. We will begin with the training sessions and posttraining tests in November 2026. We expect to complete data collection by December 2026 and finish analyzing by February 2027. Consequently, we expect to publish the results in April 2027.

**Conclusions:**

By integrating technical performance with cognitive, motivational, and perceived learning outcomes, this study will clarify not only whether but also how immersive 3D simulation can support and prepare novice trainees for physical simulator training in endourological laser-based procedures. Furthermore, it aims to inform the evidence-based development of hybrid, simulation-based curricula in medical education.

## Introduction

### Background and Rationale

Simulation-based learning (SBL) has become an important component of medical training [1,2]. By providing a safe, controlled environment that emulates authentic clinical scenarios [3], SBL allows trainees to actively and repeatedly practice procedures without risk to patients, enabling early error recognition and correction through structured feedback [4]. Furthermore, trainees can practice soft skills such as clinical reasoning (decision-making) [5], collaborative problem-solving, and interprofessional and interdisciplinary communication [6,7]. Overall, SBL has been shown to be very effective and a valuable addition to traditional training (“see one, do one, teach one”) [8].

Several simulation modalities are currently used in medical training. Among these, immersive 3D simulators, which include, for example, immersive virtual reality (VR), have gained increasing attention, although these systems vary considerably in their degree of immersion, ranging from screen-based 3D interfaces to fully immersive head-mounted VR. Immersive 3D simulation training can enhance procedural skills and facilitate their transfer, with some evidence suggesting reduced overall error rates [9-11]. Additionally, a valuable feature of immersive 3D simulators is the availability of integrated feedback and evaluation tools [12]. Despite these advantages, immersive 3D simulators are limited by their lack of realistic haptic feedback. Physical simulators such as bench-top or 3D-printed models address this limitation by providing essential haptic feedback and allowing trainees to use real surgical instruments [13]. However, the procurement and maintenance of physical simulators can be labor-intensive and time-consuming [4]. Furthermore, spontaneous training is challenging to achieve with physical simulators, in contrast to immersive 3D simulation, which can be readily accessible and straightforward to operate without assistance [4,12].

Beyond technical skill acquisition, learning outcomes in SBL can be influenced by trainees’ cognitive motivation regarding the training modality [14]. One important factor is cognitive load, defined as the mental effort imposed on working memory during learning. According to cognitive load theory, excessive sensory richness or task complexity, such as that encountered in immersive 3D environments, may increase extraneous cognitive load and hinder learning if not appropriately designed [15]. Furthermore, the trainees’ perception of the relevance and utility value of the training modality can affect their motivation and performance [16,17].

Direct comparative evidence between immersive 3D and physical simulation remains limited and often focuses narrowly on procedural skill outcomes. In the context of urology, a recent review comparing VR simulation and physical simulations across endourology, laparoscopic, robotic, and other urological procedures concluded that both modalities offer complementary strengths and recommend hybrid or multimodal curricula that combine both modalities [12].

To the best of our knowledge, no randomized controlled trials have directly compared immersive 3D simulation and bench-top models for training in endourological laser procedures. Addressing this gap, we aim to conduct a randomized controlled trial comparing immersive 3D simulation with bench-top simulation for novice trainees learning endourological laser-based procedures. By evaluating procedural performance alongside cognitive load, motivation, and perceived learning, this study seeks to generate evidence to inform the design of future endourology training curricula.

### Objectives

#### Overview

The overall aim of this randomized controlled trial is to evaluate whether immersive 3D simulation can serve as a complementary or preparatory modality to physical simulation training in teaching novice trainees endourological laser-based procedures. Specifically, this study investigates whether pretraining on an immersive 3D simulator facilitates the transfer of foundational procedural knowledge and technical skills, thereby reducing the time required to reach proficiency on the physical simulator and, subsequently, in clinical practice.

The research question is as follows: does pretraining with an immersive 3D simulator, compared to physical simulator training alone, lead to differences in posttraining technical performance among novice learners acquiring endourological laser-based procedural skills?

#### Primary Objectives

The primary objective of this study is to determine whether immersive 3D simulation differs from physical simulator training in its effects on posttraining technical performance during a standardized posttraining endourological laser-based simulation. Technical performance is defined as the ability to complete predefined endourological laser-based tasks efficiently, safely, and with adequate technical skills.

- Novice trainees who undergo physical simulator training (group A) will demonstrate superior posttraining technical performance on the physical simulator compared with trainees who undergo immersive 3D simulation training (group B), with the performance of group B expected to approach that of group A (hypothesis 1a).
- Posttraining technical performance on the physical simulator in trainees who underwent immersive 3D simulation training (group B) will exceed the technical performance observed during physical simulation training (group A) itself, reflecting a transfer of acquired skills to the physical simulator context (hypothesis 1b).

#### Secondary Objectives

The secondary objective of this study is to compare the cognitive load, intrinsic motivation, and perceived learning experienced during immersive 3D simulation to those experienced during physical simulator training.

- Immersive 3D simulation will be associated with higher perceived intrinsic cognitive load compared with physical simulation in novice trainees (hypothesis 2).
- Immersive 3D simulation will be associated with lower perceived extraneous cognitive load compared with physical simulation in novice trainees (hypothesis 3).
- Immersive 3D simulation will result in different intrinsic motivation compared with physical simulator training (hypothesis 4).
- Trainees undergoing immersive 3D simulation will report different perceived learning compared to those undergoing physical simulator training (hypothesis 5).
- Higher perceived intrinsic and extraneous cognitive load will be negatively associated with posttraining technical performance regardless of simulation modality (hypothesis 6).
- Higher intrinsic motivation will be positively associated with posttraining technical performance for both simulation modalities (hypothesis 7).
- Higher perceived learning will be positively associated with posttraining technical performance across both simulation modalities (hypothesis 8).

## Methods

### Study Design and Setting

We will conduct a single-center, 2-group, randomized controlled trial to compare the effectiveness of immersive 3D simulation vs a physical simulator in training novice learners in the laser-based endourological procedure of renal lithotripsy. In this study, “immersive 3D simulation” refers to a primarily screen-based, PC- or tablet-delivered 3D simulator with optional VR components for selected training steps (see the Study Materials section). Laser-based interventions are high-risk procedures; therefore, identifying the most effective simulation training modality in this field is crucial [18]. Furthermore, laser-based procedures offer objective and quantitative metrics for assessment, with direct visible effects [18].

Participants will be undergraduate medical students. After enrollment and completion of a pretraining baseline questionnaire, the participants will be randomly allocated to 1 of 2 parallel groups. One group will train using an immersive 3D simulation, whereas the other will train using the physical simulator, and both will undergo 1 training session. Finally, both groups will complete a standardized posttraining test on the physical simulator (Quantitative Surgical GmbH [19]).

This will be a single-center study conducted in a university setting. It will involve facilities (spacious, laser-safe room) and materials (eg, endoscopy tower, laser tower) to meet the requirements for the immersive 3D simulation and physical simulator.

This protocol was prepared in accordance with the SPIRIT (Standard Protocol Items: Recommendations for Interventional Trials) 2025 guidelines. The completed SPIRIT checklist is provided as Checklist 1.

### Participants and Recruitment

#### Overview

Participants will comprise medical students currently engaged in the clinical phase of their undergraduate medical studies. In Germany, medical studies are divided into 2 distinct phases. The first, preclinical phase spans 4 semesters and culminates in the first state examination. The second, clinical phase encompasses the remaining semesters of the program.

#### Inclusion Criteria

Individuals eligible for consideration will be undergraduate medical students who are enrolled in an undergraduate degree program in medicine and are currently in the clinical phase. Furthermore, participants should be willing to take part in and available for the required number of sessions.

#### Exclusion Criteria

To eliminate bias through previous exposure, individuals with prior training or who have previously assisted with urological endoscopic procedures will be excluded. The presence of either hearing or visual impairments is an exclusion criterion as both are needed for understanding and executing the immersive 3D simulation and the physical simulator.

#### Sample Size Calculation

We performed an a priori power analysis in G*Power (version 3.1.13) for an independent-sample *t* test [20]. As no pilot data were available and the existing literature comparing immersive 3D and physical simulation modalities is too heterogeneous in terms of outcome measures, tasks, and populations to provide a reliable empirical effect size estimate, we based our sample size calculation on a pragmatic justification rather than an empirically derived effect size. Given the substantial investment in development, licensing, and maintenance associated with immersive 3D simulation relative to established physical simulators, we reasoned that only an effect of at least medium magnitude would be sufficient to justify the additional cost and implementation effort in practice. We therefore powered the study to detect a medium effect size of *d*=0.5 using α=.05 and power (1 − β)=0.80. The analysis indicated a required sample of approximately 64 participants. Considering potential dropouts or missing data, we plan to recruit 70 participants in total.

To achieve this number of participants, a recruitment strategy will be implemented targeting individuals currently in the clinical phase of medical studies at Ludwig-Maximilians-Universität (LMU) Munich and Technical University of Munich (TUM). We will design and distribute posters in the respective teaching hospitals, as well as in their respective lecture tracts. The aforementioned posters will be modified for social media posts, which the medical faculties’ student unions will be requested to share via their social media channels (eg, Telegram and Instagram). To encourage participation, a predetermined number of vouchers for various retailers will be raffled.

### Study Materials

#### Immersive 3D Simulation

##### Overview

The training intervention will be delivered via an immersive 3D simulator developed by medverse GmbH, accessed by participants via a web link on a PC or tablet. This browser-based deployment enables scalable and flexible delivery without requiring dedicated hardware installation. Optional VR components are available for training steps that benefit from increased spatial immersion, but the primary modality is screen-based. The simulator was developed using the Unity engine (Unity Technologies), which supports immersive, interactive, and gamified training environments, enabling real-time interaction, modular scenario design, and standardized delivery of clinical workflows.

##### User Interaction

Participants navigate the simulation using standard PC or tablet input (mouse, keyboard, or touch screen). The simulator presents the endoscopic surgical field from a first-person perspective, and participants interact with anatomical structures and instruments through the interface to complete predefined procedural tasks reflecting the renal lithotripsy workflow. A representative screenshot of the simulation environment is provided in Figure 1.

**Figure 1.**
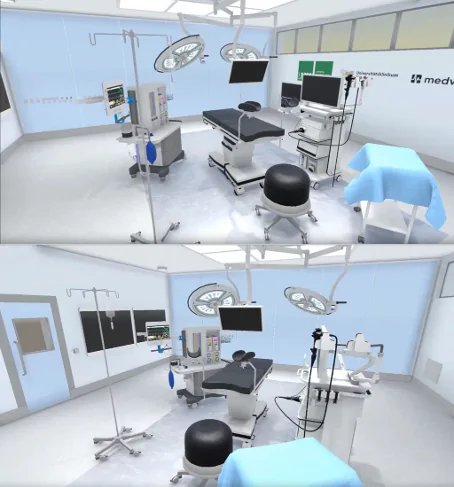
Representative screenshot of the immersive 3D simulation environment depicting the virtual operating room as rendered by the medverse GmbH simulator.

##### Feedback Mechanisms

The simulator provides multimodal, automated, real-time feedback throughout the training. This includes visual cues indicating correct or incorrect actions, audio cues accompanying key interaction events, voice-overs guiding participants through procedural steps, tooltips providing contextual information on instruments and anatomy, step progression indicators showing advancement through the task, guiding lines supporting instrument navigation, and ghost hands and objects demonstrating target movements and positions. No evaluative feedback is provided by the trainer during the training session.

##### Training Content

The training content is organized into five modules reflecting the clinical workflow of urologic endoscopic procedures: (1) preparation (device setup), (2) procedure execution (motor handling and navigation), (3) diagnostics (identification and interpretation of findings), (4) therapy (execution of therapeutic steps), and (5) postprocedure module (documentation).

##### Performance Metrics and Assessment

Training performance will be evaluated using automatically recorded simulator metrics and knowledge-based assessments. Recorded key performance indicators may encompass but are not limited to the following: time to completion, number of interactions per step, knowledge check scores, and task and module completion rates. Short knowledge check questions will follow each module. After completing the full training, participants will complete a final questionnaire assessing perceived competence, confidence, and self-reported skill acquisition.

### Physical Simulator

The physical simulator that will be used in this study was developed by Quantitative Surgical GmbH [19] with a focus on endourological procedures. The simulator consists of a replication of the male urogenital tract. This includes the penis, urethra, bladder, ureter, and kidneys. A hydrogel-based phantom mimicking the prostate, intended to mimic benign prostatic hyperplasia tissue, can be placed into the simulator. The phantom comprises an outer white layer with higher elasticity, which mimics the physiological peripheral zone of the prostate, and an inner pink layer with lower elasticity, representing the inflamed benign prostate hyperplasia tissue. Standard endourological instruments can be used with this simulator, including rigid and flexible endoscopes (ie, cysto-urethroscopes and uretero-renoscopes), and the flow of saline solution is ensured.

### Outcome Measurements

We will ask participants to complete a pretraining baseline questionnaire to ascertain demographic data (eg, age and gender), current semester, prior medical internships, and prior encounters with endoscopy (eg, observation of a urological endoscopy, the number of such encounters, and the role of the participant during the procedures). Finally, participants will be asked to create a pseudonym (eg, first 2 letters of their mother’s first name+first 2 letters of their father’s first name+their mother’s birth month+their father’s birth month).

#### Technical Performance

The technical performance will encompass the ability to complete predefined endourological tasks efficiently, safely, and with adequate technical skills. These will be measured during a posttraining test conducted on the physical simulator. We will measure global technical skill using a structured global technical skill rating scale adapted from the objective structured assessment of technical skills, which has been validated (construct and internal validity) for endourological procedures (cystoscopy and ureteroscopy) [21,22]. Domains will include respect for tissue, time and motion, instrument handling, knowledge of instruments, flow of procedure and forward planning, anatomical orientation, and knowledge of the procedure and will be rated using a 5-point Likert scale (ranging from 1=“poor” to 5=“excellent”) [22]. Furthermore, we will use critical errors as indicators of safety. The critical errors will be clearly defined in advance and will be counted using a predefined checklist. Examples of critical errors are incorrect laser activation, loss of anatomical orientation requiring repositioning, or tissue damage. Two independent experts in endourology who will be blinded to group allocation will separately assess global technical skills and safety. Disagreements between the 2 experts will be resolved by calculating the mean of their respective scores. In addition, we will use time to task completion to assess efficacy. This captures procedural flow and fluency. We will record the number of seconds and minutes from start to completion of the predefined task (eg, using a stopwatch).

#### Motivation

We will assess participants’ intrinsic motivation using items from the Intrinsic Motivation Inventory (IMI) [23]. The IMI is a well-established instrument for the evaluation of subjective motivation in experimental tasks. The present study will concentrate on the interest/enjoyment and effort/importance subscales of the IMI as these are the most pertinent to the current investigation. The interest/enjoyment scale is a 7-statement metric that quantifies the level of enjoyment or engagement that the participant experiences in relation to the activity (a self-report proxy for intrinsic motivation). The effort/importance scale measures the extent of effort exerted by the participant and their perception of the importance of achieving success. This scale uses a set of 6 statements to assess these dimensions. Each statement uses a 7-point Likert scale ranging from “strongly disagree” to “strongly agree.”

#### Cognitive Load

We will use a questionnaire to assess the cognitive load experienced by participants during training [24]. Three distinct types of load are measured using this questionnaire: intrinsic load (the inherent difficulty of the content), extraneous load (the load imposed by the way in which information is presented), and germane load (the effort invested in meaningful learning). We will use a modified version of the scale, with a total of 8 statements rated on a 7-point Likert scale ranging from “not at all true” to “completely true.”

#### Perceived Learning

Participants will evaluate their learning experience by answering 3 custom items: “Overall, I was satisfied with my learning experience,” “This type of learning met my needs as a learner,” and “I could learn well with the material presented.” The responses will be on a 7-point Likert scale ranging from “strongly disagree” to “strongly agree.” These items were adapted from previous studies on perceived learning.

### Ethical Considerations

This study was submitted to the Ethics Committee of the Faculty of Medicine at LMU Munich (26-0778).

#### Trial Registration

This trial is registered with the German Clinical Trials Register (DRKS00041224; registered on July 30, 2026).

#### Informed Consent

We will provide potential participants with enough information to enable them to provide informed consent. This includes informing them that their involvement in the study is voluntary and that they can withdraw at any time without needing to justify their decision. Subsequently, we will obtain participants’ written informed consent.

#### Privacy and Confidentiality

The data will be pseudonymized using a code, as explained above. Only the participants themselves will be able to identify their codes.

#### Participant Compensation Details

The participants will not receive any form of compensation.

### Procedure

The total duration of the study is estimated to be 2 hours, including 1 training session and 1 posttraining test.

#### Standardized Pretraining Course (75 Minutes)

##### Knowledge Module (30 Minutes)

Prior to the training sessions, participants will complete an informative online course covering the clinical and procedural fundamentals of renal lithotripsy. Content includes relevant anatomy (urethra, prostate, and bladder), indications, and intra- and postoperative risks, as well as required instrumentation and a step-by-step procedural tutorial. This module is self-paced and may be completed at any point before the training session.

##### Laser Safety Instruction Module (45 Minutes)

All participants will be provided with compulsory comprehensive training and instruction on laser safety in accordance with the Technical Rules for the Occupational Safety and Health Ordinance on Artificial Optical Radiation, the German Occupational Safety and Health Act (§12), and the Occupational Safety and Health Ordinance on Artificial Optical Radiation. It is designed to equip individuals with the necessary skills and knowledge to safely operate the equipment and access the designated rooms. This module is delivered online on a scheduled synchronous date before training sessions begin and is overseen by a certified laser safety officer.

### Training Session and Posttraining Assessment

#### Enrollment and Baseline Questionnaire (15 Minutes)

The supervisors will provide the participants with information regarding the study and data management and will then collect their informed consent. Subsequently, the participants will answer the baseline questionnaire.

#### Randomization (5 Minutes)

Following the completion of the baseline questionnaire, participants will be randomly allocated to the immersive 3D simulation group or the physical simulation group using computer-generated randomization, with sequence generation performed prior to data collection using validated randomization software and a 1:1 allocation ratio. Full blinding of participants is not feasible given that group assignment is inherent to the nature of the intervention. However, blinding will be maintained at the level of outcome assessment. A member of the research team, independent of group allocation and data collection, will assess the outcome in the posttraining test.

#### Training (45-60 Minutes)

The training sessions will be conducted in the group constellations that are allocated during randomization. To ensure comparability, both groups will practice the same predefined procedural tasks reflecting the workflow of the target procedures. Task difficulty will be held constant across groups through the use of standardized training scenarios and equivalent phantom materials for the physical simulator.

The total training time is standardized at 45 minutes across both groups. Participants in the immersive 3D simulation group will complete 3 simulation rounds, in which they will perform predefined training tasks (simulating steps of the target endourological procedures) using individual PCs or tablets and, if applicable, VR headsets. The duration of each simulation round is set to be 15 minutes, with brief breaks in between. Participants in the physical simulator group will undergo 1 simulation round lasting 45 minutes, during which they will also complete the same predefined tasks.

Trainer support will be kept equivalent for both groups. A member of the research team will be present throughout all training sessions to provide technical assistance and answer procedural questions but will not provide performance-directed feedback or coaching beyond what is protocol defined.

Participants in the immersive 3D simulation group will receive automated, real-time performance feedback integrated within the simulator, including interaction with tissue. Participants in the physical simulation group will not receive automated feedback during training; performance will instead be observable through direct engagement with the physical model and instrument handling. Neither group will receive evaluative feedback during the training session.

Following the training sessions, participants will complete the posttraining questionnaire, which will assess cognitive load, intrinsic motivation, and perceived learning.

#### Posttraining Assessment (45 Minutes)

Subsequent to the training session, there will be a break before proceeding to the posttraining test on the physical simulator for both groups. The posttraining test will be recorded. Completing the posttraining test will mark the end of participation.

### Statistical Analysis

#### Descriptive Statistics

We will use descriptive statistics to summarize participant demographics, baseline questionnaire data, posttraining questionnaire data, and all outcome measures.

For *continuous data* such as age, times, and scale values, we will calculate means and SDs. For *categorical data* such as gender, we will calculate counts and percentages.

#### Assumption Checks

Prior to conducting inferential tests, we will assess the assumptions underlying the independent-sample *t* test. Normality of the outcome variables within each group will be evaluated using the Shapiro-Wilk test and visual inspection of *Q*-*Q* plots. Homogeneity of variance across groups will be assessed using the Levene test. Where the assumption of normality is violated, we will use the Mann-Whitney *U* test as a nonparametric alternative to the *t* test. Where the assumption of equal variances is violated but normality holds, we will apply the Welch *t* test instead of the standard Student *t* test.

#### Missing Data Handling

We will examine the pattern and extent of missing data for all outcome variables. The mechanism of missingness will be assessed using the Little missing completely at random test. If data are missing completely at random or at random, multiple imputation (m=20 imputed datasets using predictive mean matching) will be used to handle missing values, and results will be pooled using the Rubin rules. A complete-case analysis will be reported alongside the imputed analysis as a sensitivity check. The amount and pattern of missing data will be reported transparently in the Results section.

#### Primary Hypothesis Testing

To test hypotheses 1a and 1b, we will use independent-sample *t* tests (or the nonparametric or Welch-corrected alternative, as appropriate) to compare posttraining technical performance between the immersive 3D simulation group and the physical simulator group. As the primary hypothesis, this comparison will not be adjusted for multiple testing.

#### Secondary Hypothesis Testing

To test hypotheses 2 to 5, group differences in intrinsic cognitive load, extraneous cognitive load, intrinsic motivation, and perceived learning will be analyzed using independent-sample *t* tests (or the corresponding nonparametric or Welch-corrected alternative). As these 4 comparisons test related secondary outcomes, we will apply a Holm-Bonferroni correction to control the familywise error rate.

To test hypotheses 6 to 8, which concern associations among cognitive load, intrinsic motivation, perceived learning, and posttraining technical performance, we will conduct multiple linear regression analyses with technical performance as the dependent variable and the respective predictor (cognitive load, motivation, or perceived learning) entered alongside group condition as a covariate. To examine whether these associations differ by simulation modality, we will additionally test the predictor by group interaction term in an exploratory moderation analysis. Standardized regression coefficients, 95% CIs, and model *R*^2^ will be reported.

All analyses will be conducted in R (version to be specified at the time of analysis; R Foundation for Statistical Computing), and the significance threshold will be set at α=.05 unless adjusted as described above.

## Results

The study is funded between September 2025 and August 2027. Recruitment will start in September 2026. We will begin with the training sessions and posttraining tests in November 2026. We expect to complete data collection by December 2026 and finish analyzing by February 2027. Consequently, we expect to publish the results in April 2027.

## Discussion

### Expected Findings

This randomized controlled trial protocol describes a study designed to evaluate whether immersive 3D simulation can serve as a complementary or preparatory modality to physical simulation training in laser-based endourological procedures among novice trainees. We hypothesize that pretraining on the immersive 3D simulator will facilitate the transfer of procedural knowledge and technical skills, thereby accelerating proficiency on the physical simulator. Beyond technical performance, we anticipate that the two modalities will differ in their effects on cognitive load, intrinsic motivation, and perceived learning and that these factors will, in turn, moderate technical skill acquisition. Specifically, we expect that immersive 3D simulation will be associated with higher intrinsic and lower extraneous cognitive load and that higher intrinsic motivation and perceived learning will be positively associated with posttraining performance across both modalities.

This study contributes to a limited but growing body of comparative evidence on simulation modalities in urological training. A recent review concluded that immersive 3D and physical simulation offer complementary strengths and recommended hybrid or multimodal curricula combining both approaches [12]. Our study directly responds to this recommendation by empirically testing whether immersive 3D simulation can function as a preparatory complement to physical simulation. Furthermore, by incorporating cognitive load, intrinsic motivation, and perceived learning, this study recognizes that learning outcomes are shaped not only by what trainees practice but also by how they cognitively and motivationally engage with the training modality [14-17]. This could deliver information relevant to educational theory and inform the selection or blending of simulation modalities, providing guidance for curriculum design and educational resource allocation.

Should the findings support the effectiveness of immersive 3D simulation as a preparatory modality, an important next step could be the development of a standardized simulation curriculum for endourological laser-based procedures, integrating both modalities in a sequenced, hybrid format. A particularly promising avenue would be the formalization of such a curriculum as an entrustable professional activity, defining the competencies, milestones, and assessment standards required for trainees to be entrusted with these procedures with decreasing supervision [25]. To date, no such entrustable professional activity exists for endourological laser procedures, representing a significant gap in urological training frameworks that future work should seek to address.

Among the strengths of this study is its two-group randomized controlled trial design, with stratified randomization ensuring balanced group composition and supporting the comparability of findings, and its standardized training exposure across both groups, which improves comparability between them. Furthermore, we will incorporate blinded expert assessment of the participants’ technical performance, eliminating bias, and use validated measurement instruments for the secondary outcomes.

Nevertheless, several limitations warrant consideration. The single-center design may limit the study’s generalizability. Furthermore, the study population of clinical-phase medical students may not represent more experienced trainees, and findings might not directly translate to clinical practice. Finally, the study also assesses short-term performance only; longer-term retention and clinical transfer remain beyond its scope. However, currently, there is an absence of a standardized training framework and schedule for endourological procedures in Germany. We hope that the present study has the potential to contribute to the advancement of the development of standardized, simulation-based training, thereby addressing one part of this gap.

### Conclusions

SBL is increasingly recognized as an essential component of modern medical education, yet the comparative evidence base for specific modalities in procedural specialties such as endourology remains sparse. By directly comparing immersive 3D and physical simulation in the context of laser-based endourological procedures and integrating technical, cognitive, and motivational outcomes within a single rigorous trial, this study is positioned to make a meaningful contribution to this evidence base. The findings could encourage evidence-based, hybrid training pathways in simulation curricula design tailored to the demands of high-risk procedural learning. Moreover, this study represents a step toward a long-term goal of establishing standardized, competency-based training frameworks for endourological procedures.

## Supplementary material

10.2196/94628 Checklist 1 SPIRIT 2025 checklist.

## Abbreviations

IMI: Intrinsic Motivation Inventory
LMU: Ludwig-Maximilians-Universität
SBL: simulation-based learning
SPIRIT: Standard Protocol Items: Recommendations for Interventional Trials
TUM: Technical University of Munich
VR: virtual reality
